# Antimicrobial pharmacokinetics in pediatric patients on kidney replacement therapy: a comprehensive narrative review

**DOI:** 10.1007/s00467-025-07083-8

**Published:** 2025-12-06

**Authors:** Shannon Reinert, Sonya Tang Girdwood, H. Rhodes Hambrick

**Affiliations:** 1 Division of Nephrology and Hypertension, Cincinnati Children’s Hospital Medical Center, Cincinnati, OH USA; 2 Division of Hospital Medicine, Division of Translational and Clinical Pharmacology, Center for Acute Care Nephrology, Cincinnati Children’s Hospital Medical Center, Cincinnati, USA; 3 Division of Nephrology, Ann and Robert H. Lurie Children’s Hospital of Chicago, Chicago, IL USA; 4 Department of Pediatrics, Northwestern University Feinberg School of Medicine, Chicago, IL USA; 5 Department of Pediatrics, University of Cincinnati College of Medicine, Cincinnati, OH USA

**Keywords:** Pharmacokinetics, Pediatrics, Dialysis, Kidney replacement therapy, Antimicrobials, Antibiotics

## Abstract

Patients on dialysis frequently require antimicrobial administration for a variety of reasons, including sepsis. Studies in adults have demonstrated a need for antimicrobial dosing adjustment in patients on dialysis due to altered pharmacokinetics (PK) as compared with patients not on kidney replacement therapy (KRT). High-quality studies evaluating PK of frequently used antimicrobials in pediatric patients on KRT are lacking. Dosing recommendations in this population are extrapolated from adult studies, which relies on the assumption that PK between adult and pediatric patients are similar. We conducted a literature review to describe the existing literature on antimicrobial PK in pediatric patients on dialysis and outline gaps that should be the focus of future research. We identified 56 original studies evaluating PK of twelve different antimicrobials in pediatric patients on hemodialysis, peritoneal dialysis, or continuous KRT. This narrative review demonstrates that antimicrobial PK in pediatric patients on dialysis are understudied, despite this population being at increased risk of infection and vulnerable to drug toxicity. Future research should focus on drugs that are most used and drugs with known/suspected risk of toxicity at elevated concentrations.

**Graphical Abstract:**

**Figure:**
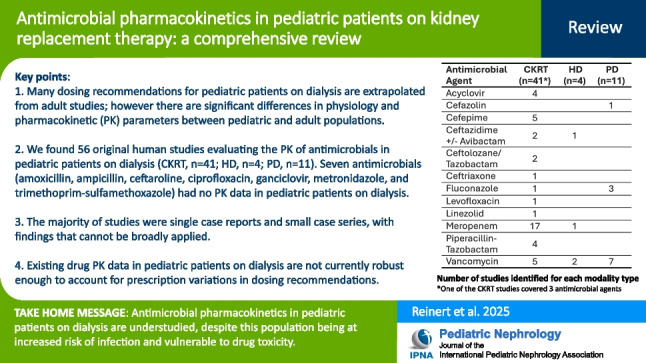
A higher resolution version of the Graphical abstract is available as Supplementary information

**Supplementary Information:**

The online version contains supplementary material available at 10.1007/s00467-025-07083-8.

## Introduction

In 2022, there were 1379 pediatric patients receiving chronic dialysis, with 734 on peritoneal dialysis (PD) and 645 on hemodialysis (HD) [1]. Furthermore, critically ill children, including those with sepsis, are at risk for severe acute kidney injury (AKI) and sometimes require kidney replacement therapy (KRT) [2]. PD is also frequently used for the management of AKI and fluid overload after cardiopulmonary bypass in infants [3]. Regardless of modality, patients on dialysis have an increased risk of infections. Patients with chronic kidney disease (CKD) have a degree of immune dysfunction [4]. Additionally, HD and PD catheters increase the infection risk both from violation of the skin barrier and from the presence of plastic within the body [5, 6]. Consequently, bacterial infections are one of the leading causes of hospitalization and mortality in patients receiving chronic dialysis [7]. Furthermore, critically ill patients with sepsis-associated AKI sometimes require acute dialysis [8]. Taken together, timely antimicrobial administration is crucial for the adequate treatment of sepsis in this vulnerable population, regardless of dialysis type [9].

Not only is timely administration important, but accurate dosing of antibiotics in patients receiving dialysis is critical. Specifically, inadequate antimicrobial exposure due to suboptimal dosing can lead to inadequately treated infections and emergence of antimicrobial resistance. Insufficient antimicrobial coverage can lead to persistent bacteremia or peritonitis and the need for catheter (HD or PD) removal. Conversely, excessive antimicrobial exposure puts patients at risk for toxicity. For example, supratherapeutic vancomycin exposure puts patients at risk for ototoxicity, nephrotoxicity, and vancomycin flushing syndrome [10], while high cefepime exposure has been reported in association with neurotoxicity in children with kidney impairment [11, 12].

Studies in adults have demonstrated a need for antimicrobial dosing adjustment in patients on dialysis due to altered PK as compared with patients not on KRT [13–16]. The most obvious PK change is drug clearance (CL) due to extracorporeal CL in HD/CKRT (continuous KRT) and drug losses in the effluent in PD. Additionally, studies have demonstrated that CKD and dialysis can affect non-kidney CL [14, 17] and that the volume of distribution (Vd) can be affected by CKD, AKI, fluid status, and critical illness [13, 15, 16]. The impact of KRT on a drug’s PK depends on several factors including drug-related characteristics (e.g., molecular weight (MW), degree of protein binding, and Vd), dialysis prescription, and the clinical situation (e.g., critical illness or CKD) [13, 15, 16]. High-quality studies evaluating the PK of frequently used antimicrobials in pediatric patients on KRT are lacking. Many dosing recommendations in this population are extrapolated from adult studies. While this extrapolation relies on the assumption that the PK between adult and pediatric populations are similar, there are significant differences in physiology and pharmacokinetic parameters [18, 19], particularly in infants and neonates [20]. Some of these differences are due to changes in body composition that occur throughout childhood. For example, the ratio of total body surface area to body mass in infants and young children far exceeds that in adults [20]. Infants in the first 6 months of life have significantly expanded total-body-water and extracellular water as compared with older children and adults, affecting Vd, and can lead to lower plasma concentrations of drugs when they are administered in a weight-based fashion. Finally, there is significant maturation in both liver, kidney, and gastrointestinal function (i.e., ontogeny) in the first few months after birth that continues to some extent well into childhood [20]. There are numerous examples when extrapolating pediatric dosing recommendations from purely adult PK data, without considering these physiologic differences, would result in inaccurate dosing [21]. The aim of this narrative review is to describe the existing literature on antimicrobial PK in pediatric patients on dialysis and outline gaps that should be the focus of future research.

## Literature search/methodology

An international group of experts in extracorporeal life support (ECLS, including extracorporeal membrane oxygenation (ECMO) and CKRT) convened in 2022 to address existing gaps in the field of PK for patients receiving ECLS and formulated a list of high-priority drugs for future research [22]. Antimicrobials for this narrative were chosen from the drugs from this list alongside other antimicrobials frequently used in children receiving HD and PD.

This review is based on an extensive search of original peer-reviewed literature. We conducted a PubMed search in June 2025 for literature with no language or time limitations. Search items included “pediatric”, “pharmacokinetics”, “peritoneal dialysis”, “hemodialysis”, “continuous kidney replacement therapy,” “continuous renal replacement therapy,” and “dialysis” along with the antimicrobial of interest. Relevant articles referenced in publications identified from our search were also reviewed. Prospective and retrospective pharmacokinetic studies, case series, case reports, or conference abstracts reporting on patients 25 years or younger (hereafter referred to as “pediatric patients”) and receiving dialysis were included. When appropriate, dosing recommendations were compared to those from a popular tertiary dosing reference (Lexi-Comp Online database) and the International Society of Peritoneal Dialysis (ISPD) guidelines.

## Results

For this review, 56 original human studies were considered relevant. We structured the literature findings according to dialysis modality (CKRT (*n* = 41), HD (*n* = 4), and PD (*n* = 11)) and by antimicrobial agent (acyclovir (*n* = 4), amoxicillin (*n* = 0), ampicillin (*n* = 0), cefazolin (*n* = 1), cefepime (*n* = 5), ceftaroline (*n* = 0), ceftazidime ± avibactam (*n* = 3), ceftolozane/tazobactam (n = 2), ceftriaxone (n = 1), ciprofloxacin (n = 0), fluconazole (n = 4), ganciclovir (*n* = 0), levofloxacin (*n* = 1), linezolid (*n* = 1), meropenem (*n* = 18), metronidazole (*n* = 0), piperacillin-tazobactam (*n* = 4), trimethoprim-sulfamethoxazole (*n* = 0), and vancomycin (*n* = 14)).

## Continuous kidney replacement therapy (CKRT)

The modalities in common use in CKRT include continuous veno-venous hemofiltration (CVVH), continuous veno-venous hemodialysis (CVVHD), and continuous veno-venous hemodiafiltration (CVVHDF), with CCVHDF most used in children [23]. These modalities remove solutes via diffusive and/or convective CL [22]. Drugs with a smaller Vd and lower MW are most efficiently removed by CKRT. Drug CL is significantly affected by modality and CKRT intensity, primarily by the effluent flow rate (Qef). This includes the ultrafiltration rate, which contributes to convective CL. Qef may vary based on the indication for CKRT (e.g., high Qef for ammonia clearance in patients with inborn errors of metabolism), but data are limited regarding the impact of changes in Qef on drug PK.

Additionally, critically ill patients on CKRT may receive additional extracorporeal therapies such as ECMO, the molecular absorbent recirculating system (MARS), and plasmapheresis, which can further alter Vd and CL [22]. Finally, patients on CKRT are typically critically ill, and drug PK in critically ill patients can be affected by additional variables such as concomitant medications (etc. inotropes/vasopressors), inflammation, and albumin concentrations [24].

### Beta-lactam antibiotics

The efficacy of beta-lactam antibiotics is dependent on the time that free (non-protein bound) concentrations are above the bacterial minimum inhibitory concentration (*f*T > MIC) [25]. Beta-lactam concentrations are not typically measured, so the risk of inadequate target attainment and toxicity is understudied, especially in patients on CKRT.

#### Cefepime

Cefepime has low MW and a low degree of protein binding (20%), making it susceptible to CKRT CL [26]. Five studies evaluated cefepime PK in a total of 14 pediatric patients on CKRT. One study developed a population PK model (PopPK) in 17 patients on ECMO, two of whom were also on CKRT [27]. Population PK is the study of how concentrations vary over time among a group of patients (i.e., population). A PopPK model is developed from observed drug concentrations, dosing regimens and relevant patient and clinical factors from a group of patients to describe the typical PK parameters (e.g., CL, Vd) in that population and the variability that can exist within and among individuals [28]. Once a PopPK model is created, it can be used to guide initial drug dosing for future patients, based on variables such as age, size, clinical status, and kidney function, through Monte Carlo simulations (i.e., virtual clinical trial simulations) [29]. This study was focused on the effect of ECMO on cefepime CL and surprisingly the two patients on CKRT had a decrease in CL of 40% compared to those on ECMO alone. Unfortunately, limited information about kidney function was provided for these two patients and it is likely that CKRT was not the main contributor to lower CL but rather intrinsic kidney injury decreasing the patient’s native CL. Overall, cefepime concentrations were not consistently adequate for the treatment of pseudomonal infections, and the authors concluded that this population would benefit from cefepime therapeutic drug monitoring (TDM) to ensure adequate drug exposure. Two studies, with 11 patients total, reported a wide variability in cefepime exposure in patients receiving a range of CVVHDF clearances [30, 31]. One of these studies specifically found a correlation between Qef and drug CL, with Qef accounting for 77% of the observed variance [31]. Another study added a CKRT module, which can account for CKRT prescription (i.e., Qef, filter size), to a previously published cefepime PopPK model for critically ill children not on CKRT [26]. The adapted PopPK model was evaluated using historical patient samples from four patients (from the study by Pavia et al. [31]). Monte Carlo simulations (MCS) of various cefepime dosing strategies were then conducted in 1000 virtual patients. Their results suggest that 4-h extended infusions or a 24-h continuous infusion (CI) would be needed to meet more stringent pharmacodynamic targets (100% *f*T > 4 × MIC) or when using high intensity CKRT. This is somewhat reflected in current Lexicomp recommendations, which recommend 50 mg/kg q8-12 h (max 2000 mg), with a caveat to consider extended infusions or more frequent administration for high effluent, bacteria with high MIC, or deep-seated infections in severely ill patients. A single case report evaluated cefepime PK in a patient on CVVHD using the Cardio-Renal Pediatric Dialysis Emergency Machine (CARPE DIEM) [32].

#### Ceftazidime ± avibactam

Ceftazidime is a small molecule with low-protein binding and a relatively small Vd [33]. Ceftazidime-avibactam is a novel cephalosporin/beta-lactamase inhibitor developed to address the recent rise in MDR-Gram-negative pathogens [34]. One case series reported ceftazidime PK in three critically ill children who received hemoadsorption in combination with CKRT. This study focused mostly on the effect of hemoadsorption on drug CL and found that hemoadsorption contributed to extracorporeal CL in addition to the hemofilter. Despite this increased CL, the plasma concentrations were adequate to reach the pharmacodynamic target (4–6 × MIC) using a dosing regimen of 50 mg/kg q12h [35]. A case study of ceftazidime-avibactam found that a 2-h infusion of 30–7.5 mg/kg every 8 h did not reach the PK/pharmacodynamic targets (100% *f*_T_ > 4 × MIC), and CL was found to be higher than that reported in the adult literature [34]. Given the sparsity of data available for pediatrics on dialysis, there are no pediatric-specific dosing recommendations available at the time of this publication for patients on dialysis.

#### Ceftolozane-tazobactam

Ceftolozane is a small molecule with kidney elimination and low-protein binding [36]. Ceftolozane-tazobactam is a combination of ceftolozane and a beta-lactamase inhibitor tazobactam used to treat multi-drug resistant (MDR) *P. aeruginosa* strains [37]. Two studies evaluated ceftolozane-tazobactam PK in critically ill children on CKRT. One case report demonstrated success treating a critically ill child with disseminated MDR *P. aeruginosa* and AKI requiring CKRT using a high-dose CI of ceftolozane-tazobactam [36]. The second study evaluated ceftolozane-tazobactam PK in 3 critically ill children, one of whom was on high-effluent CKRT, and found that 30 mg/kg every 8 h was appropriate. CL from CKRT was similar in the two studies (0.02–0.06 L/kg/h [36] and 0.07 L/kg/h [37]). There are no formal pediatric-specific dosing recommendations available at the time of this publication for patients on dialysis.

#### Ceftriaxone

Ceftriaxone is highly protein bound (70–90%), meaning that it should be less susceptible to extracorporeal CL [38]. Guidelines report that ceftriaxone is not dialyzable, and no dosing adjustment is needed. One case series evaluated ceftriaxone PK in pediatric patients on CKRT [38]. Although all three patients in this study achieved their PD targets, CKRT provided 25–42% of total drug CL, suggesting some dialyzability. Since none of the patients in this study were prescribed high-effluent CKRT prescriptions, it is possible that high-effluent prescriptions may result in subtherapeutic ceftriaxone concentrations. It was concluded that for anuric pediatric patients receiving standard dose CKRT, every 24-h dosing is likely sufficient for target attainment. One patient demonstrated ceftriaxone accumulation, and simulations for this patient suggested that every 48-h dosing would be adequate.

#### Meropenem

Meropenem is a broad-spectrum carbapenem with activity against many gram-positive and gram-negative organisms, including *Pseudomonas aeruginosa*, which is often difficult to eradicate. Seventeen studies evaluated meropenem PK in children on CKRT with eight developing a PopPK model. Covariates that were found to be significant for CL in these PopPK models were weight [39–46], kidney function (not specified whether pre-CKRT or on-circuit) [40, 43], systemic inflammatory response syndrome score [40], Qef [45], and hemofilter size [39]. We were able to directly compare median CL for four of these studies [42, 44–46]. When normalized to a 70 kg patient, the median CL in these populations ranged from 4.1 to 6.82 L/h/70 kg^0.75^. It was difficult to compare CL from the other four studies, due to a lack of allometric scaling and different units and covariates used. Four studies also performed MCS using their PopPK model [40–43]. Dosing recommendations from these studies, as applicable, are detailed in Supplemental Table 1. All four studies discussed that a prolonged or CI may be needed, depending on PK/PD goals. There were nine additional case reports and case series that used various dosing strategies, as detailed in Table 1 [35, 47–54]. Lexicomp recommends 20 mg/kg infused over 1–4 h q8h, but states that drug CL depends on Qef, and higher doses may be needed when MIC > 4 mg/L, citing three of the PopPK studies discussed as rationale [42–44].

**Table 1:** Studies evaluating antimicrobial PK in pediatric patients on CKRT

Drug	Dose(s) used^a^	Modalities used	CKRT (*n*)	ECMO (*n*)	No KRT (*n*)	Age	Wt (kg)
*Beta-lactam antibiotics*							
Cefepime [26]	Six dosing strategies simulated, see ref	CVVHDF	4 patients; 1000 simulations	0	N/A	2–25 yr	
Cefepime [27]	50 mg/kg q12h and q24h	CKRT and ECMO	2	17	15	1.3–22.2 m	3.3–10
Cefepime [32]	50 mg/kg q24h then q12h when clearance increased	CARPE DIEM (CVVHD)	1	0	N/A	4 m	2.5
Cefepime [30]	50 mg/kg, ranging from q6h—q12h	CVVHDF	4	0	N/A	0.5–5 yr	5.4–25
Cefepime [31]	50 mg/kg q8h-q12h, 1 g q8h, and 2 g q12h	CVVHDF	7	0	N/A	2–20 yr	10.5–61.5
Ceftazidime [35]	50 mg/kg q12h	CVVH and CVVHDF	3	0	N/A	Median 11.5 yr (range 6.5–14.75)	Median 42 (range 26–71)
Ceftazidime-avibactam [34]	2-h infusion q8h, 30–7.5 mg/kg	CVVHDF	1	0	N/A	6 m	8
Ceftolozane-tazobactam [37]	30 mg/kg q8h	CKRT	1	0	2	9 m	5.8
Ceftolozane-tazobactam [36]	150 mg/k/d CI	CVVHDF	1	0	N/A	15 m	7
Ceftriaxone [38]	50 mg/kg (max 2 g) q12h-24 h	CVVHD and CVVHDF	3	0	N/A	2–17 yr	11.7–74.5
Meropenem [42]	Mean 30 mg/kg q12h	CVVH and CVVHDF	9 for PK, then 1000 simulations	4	N/A	0.1–18.9 yr	2.6–56.3
Meropenem [43]	20 or 40 mg/kg q8h over 60 min or over 3 h	CVVHDF, ECMO	9	13	16	Median 2 yr (IQR 0.71–3.88)	Median 11.5 (IQR 9.5–36.3)
Meropenem [41]	Off ECMO 20–40 mg/kg 2-3x/d. On ECMO, 20 mg/kg loading + CI (determined by age)	CKRT, ECMO	31	38	14	Median 3 d (IQR 0–465)	Median 7.88 (IQR 3.62–11.97)
Meropenem [40]	Median 105 mg/k/d (range 40–293). By 1- or 3-h infusion	CVVHD, CVVHDF, ECMO	8	3	26	Mean 1.4 yr (range 0.03 to 14.6)	Mean 8.9 (range 2.7 to 40.9)
Meropenem [46]	20 mg/kg q8h and q12h	CVVHD, CVVH, and CVVHDF	5,000 simulations	No	N/A	0–18 yr	Multiple, see ref
Meropenem [45]	Simulated 6 different dosing scenarios, see ref	CVVH, CVVHD, CVVHDF	27 (86 samples)	Not specified (25 samples)	N/A	Median 4 yr (IQR 0–11)	Median 16 (IQR 7–35)
Meropenem [44]	30–122 mg/kg/d (q8h or CI)	CKRT	11	8	29	Median 16.8 m (range 1.4–187.2)	Entire group median 9.1 (range 3.8–59)
Meropenem [39]	40 mg/kg q8h, spaced to q12h after 4th dose	CVVHDF	7	0	9	Median 48 m (IQR 5–106)	Median 20 (IQR 7.4–40)
Meropenem [35]	40 mg/kg q8h	CVVHDF and CVVH	5	0	N/A	Median 11.5 yr (range 6.5–14.75)	Median 42 (range 26.25–71.25), for all 10
Meropenem [47]	Mean 18.6 mg/kg	CVVH and CVVHDF	7	0	N/A	5–21 yr	21.9–72.6
Meropenem [49]	40 mg/kg bolus over 30 min followed by 10 mg/kg/h CI	CVVHDF	1	1	N/A	10 d	2.8
Meropenem [48]	40 mg/kg q8h then due to lack of response, 10 mg/kg/h CI	CVVHDF	1	1	N/A	29 d	2.5
Meropenem [51]	3 h infusion of 120–300 mg/k/day divided q8h	CVVHD and ECMO	1	1	N/A	19 m	9
Meropenem [53]	10 mg/kg/h, switched to 20 mg/kg q12h due to supratherapeutic levels	CVVHDF and ECMO	1	1	0	4 yr	NR
Meropenem [52]	300 mg daily (17.1 mg/kg)	PIKRT	1	0	N/A	5 yr	17.5
Meropenem [50]	20 mg/kg over 1 h q8h	CVVHDF and ECMO	6	6	21	Median 2 yr (IQR 1.13–6.88)	Median 12.5 (IQR 8.6–27.15)
Meropenem [54]	40 mg/kg q8h	CARPE DIEM (CVVHD)	1	0	N/A	4 d	2.57
Piperacillin [55]	Median 300 mg/k/d	CKRT	32 patients (93 samples)	Not specified (4 samples)	N/A	Median 4 yr (IQR 0.6–11)	Median 15 (IQR 6–38)
Piperacillin [56]	100 mg/kg q8h, spacing to q12h after 4th dose	CKRT	13	4	19	Median 7 m (range 3 m-15 yr)	Median 8.1 (range 4–63)
Piperacillin [57]	48 mg/kg q8h initially due to poor renal function. Received an extra 38 mg/kg, then was transitioned to adult dosing (3375 mg q6h, 80 mg/kg) while on CKRT alone	CKRT and MARS	1	0	N/A	13 yr	16.4
Piperacillin [58]	100 mg/kg q6h	CKRT	1	0	0	8 yr	23.5
*Other antibiotics*							
Levofloxacin [59]	10 mg/kg q12h	CVVH and CVVHDF	3	0	N/A	Median 11.5 yr (range 6.5–14.75)	Median 42 (range 26–71)
Linezolid [60]	Median 30 mg/kg/d	CKRT	15	2	48	Median 3.85 yr (range 0.1–15.3)	Median 15 (range 4.2–70)
Vancomycin [63]	Mean dose 14 mg/kg	CVVHDF	138	0	N/A	Median 4.9 yr (IQR 1–14.5)	Mean 31 (SD 25.8)
Vancomycin [64]	NR	CVVHD, CVVHDF, and ECMO	41 patients (279 doses)	25	N/A	Median 1.33 yr (IQR 0.096–11)	Median 9 (IQR 4.24–36)
Vancomycin [24]	15–20 mg/kg bolus followed by 18–30 mg/L in the CKRT solution	CVVH, CVVHD, and CVVHDF	11	0	N/A	0.08–18 yr	3.1–61.7
Vancomycin [65]	40 to 60 mg/kg/d divided q6h (over 2 h)	CKRT and ECMO	11	29	18	Median 2.7 yr (1 m– 14 yr)	NR
Vancomycin [61]	Median dose 20/k/d	CVVHDF	75	0	N/A	Median 2.2 yr (IQR 0.3–11.84)	Median 12.3 (IQR 4.9–27.8)
*Antivirals*							
Acyclovir [66]	500 mg/m^2^ over 1 h q8h	CVVHDF	1	0	N/A	21 m	10
Acyclovir [67]	3–10 mg/kg/d	CAVH, CAVHDF, and CVVHD	1	0	N/A	31 m	12
Acyclovir [68]	30 mg/kg q8h initially, then CI added to dialysate (5.5 mg/L) when viral load remained unchanged	CVVHD, CVVHDF, and ECMO	1	1	N/A	14 d	NR
Acyclovir [69]	20 mg/kg over 1 h q8h	CVVHDF and TPE	1	0	N/A	8 d	2.95
*Anti-fungal*							
Fluconazole [71]	10 mg/kg daily	CVVHD	1	0	N/A	17 yr	60

*CKRT*, continuous kidney replacement therapy; *ECMO*, extracorporeal membrane oxygenation; *KRT*, kidney replacement therapy; *yr*, years; *wt*, weight; *CVVHDF*, continuous veno-venous hemodiafiltration; *m*, months; *d*, day; *CAVH*, continuous arteriovenous hemofiltration; *CAVHDF*, continuous arteriovenous hemodiafiltration; *N/A*, not applicable; *CVVHD*, continuous veno-venous hemodialysis; *CI*, continuous infusion; *NR*, not reported; *TPE*, therapeutic plasma exchange; *PIKRT*, periodic intermittent kidney replacement therapy

^a^30-min infusion unless otherwise specified

#### Piperacillin

Piperacillin is usually combined with the beta-lactamase inhibitor tazobactam and commonly prescribed in the PICU due to its broad-spectrum antibacterial activity, including against *P. aeruginosa* and other MDR bacteria [55]. It is hydrophilic, has low-protein binding, and a low MW. Four studies evaluated piperacillin PK in pediatric patients on CKRT. One study (93 samples from 32 patients) developed a PopPK in critically ill children receiving CKRT and conducted MCS to determine the optimal piperacillin dosing, finding both weight and residual urine output (UOP) to be significant covariates [55]. MCS demonstrated that a CI would be necessary to attain PK targets for any patient with > 0.1 mL/kg/hr residual UOP while on CKRT. Anuric patients, on the other hand, were at risk for accumulation, and therefore benefited from a loading dose (LD) followed by intermittent (every 12 h) dosing. CKRT prescription parameters did not significantly affect CL in this study. Another group developed a PopPK model using 429 piperacillin concentrations from 32 critically ill children (13 of whom received CKRT) [56]. Significant covariates on CL included eGFR and height (kidney CL), weight (non-kidney CL), and filter surface area (CKRT CL). Based on their simulations with 1000 virtual patients, a 24-h CI (200 mg/kg/day of piperacillin) would be optimal in patients on CKRT, though 100 mg/kg every 8 h (30-min infusion) could be considered if a CI was not logistically possible. General recommendations for piperacillin dosing in pediatric patients on CKRT reflect these findings and cite this study. We were unable to directly compare the CL and Vd between these two PopPK studies given that one study used allometric scaling and the other did not. There were two case reports evaluating piperacillin PK in a pediatric patient on CKRT, in conjunction with MARS [57], and in a pediatric patient receiving high-dose CKRT [58]. These are included in Table 1.

### Other antibiotics

#### Levofloxacin

Levofloxacin is a lipophilic drug that is cleared by the kidneys with approximately 30% protein binding. The most relevant predictor of fluoroquinolone efficacy is the ratio of the 24-h area under the concentration time curve to the MIC (AUC_24_/MIC) [59]. One case series reported levofloxacin PK in three critically ill children who received hemoadsorption in combination with CKRT. This study focused mostly on the effect of hemoadsorption on drug CL. However, they reported that the plasma concentrations were adequate to reach their PK/PD target (Cmax = 10 × MIC) using a dosing regimen of 10 mg/kg q12h [35]. Lexicomp recommends 10 mg/kg every 24 h for patients receiving CKRT. Of note, Lexicomp’s adult hemodialysis section states 21% is dialyzed in a 4-h high-flux HD session based on an adult study, while the pediatric section states it is not removed by dialysis, which is inconsistent with existing data and PK principles.

#### Linezolid

Linezolid has significant hepatic CL, but the kidney is still responsible for about 35% of elimination [60]. Lipophilic drugs like linezolid tend to have larger volumes of distribution and are less accessible for removal by extracorporeal therapy. Accurate dosing is of particular importance, due to the risk of toxicity, including thrombocytopenia, anemia, and leukopenia, with elevated concentrations. One PopPK analysis evaluated linezolid PK in 63 critically ill pediatric patients, including 15 patients on CKRT. They found that standard dosing did not need to be adjusted for sensitive pathogens (MIC ≤ 1 mg/L). CKRT was analyzed as a covariate but showed no significant influence on PK parameters. Current recommendations suggest no dose adjustment for patients on CKRT.

#### Vancomycin

Vancomycin is primarily cleared by the kidney [61]. Additionally, it has a low Vd and is only 50% protein bound, which allows it to easily diffuse through dialysis membranes. Critically ill children frequently receive vancomycin for treatment of confirmed or presumed infections, particularly those due to methicillin-resistant *Staphylococcus aureus* (MRSA) [62]. In clinical practice, trough concentrations of 10–20 mg/L are commonly used; however, trough concentrations are a surrogate to estimate AUC and are often not well correlated. The bactericidal activity of vancomycin is optimal when the AUC/MIC ratio is ≥ 400 [62]. Appropriate vancomycin dosing is of particular importance for patients on CKRT for AKI, given that nephrotoxicity associated with elevated concentrations could inhibit kidney recovery.

Five studies evaluated vancomycin PK in pediatric patients on CKRT. One study developed a PopPK model for vancomycin therapy in pediatric patients on CVVHDF, with dosing recommendations to achieve an AUC/MIC ≥ 400 [63]. Total daily doses of 40–50 mg/kg/day divided every 8–12 h resulted in the highest PTA while maintaining serum trough concentrations < 20 mg/L. Several covariates were found to be significant and were retained in the final model for CL, including serum creatinine (unclear if pre-dialysis), blood urea nitrogen, dialysate flow rate (Qd), and ultrafiltration flow rate. One retrospective cohort study included 177 samples from 75 individual critically ill children on CVVHDF and reported that vancomycin concentrations significantly decreased with increased Qef, Qd, replacement fluid flow rate, and residual UOP [61]. The patients received a median of 20.11 mg/kg vancomycin per day, and over 25% of samples were subtherapeutic (< 10 mg/L). One large study analyzed 279 samples from 41 patients on CKRT and found that the median dosing regimen needed to achieve therapeutic trough concentrations was 17.5 mg/kg/dose every 12 h [64]. One case series described their experience adding vancomycin into the CKRT solution (dialysate and/or replacement solution), essentially providing CI [24]. The dialysis solution vancomycin concentrations ranged from 18 to 35 mg/L, resulting in serum concentrations ranging from 12.9 to 28.7 mg/L. Ten of the 11 patients in the study achieved the target vancomycin plateau level (> 15 mg/L) within 8 h. An additional case series reported on vancomycin PK in pediatric patients on ECMO, including 11 patients on CKRT, and found a wide variability in PK parameters [65]. The majority of these studies discussed the importance of frequent TDM in this population, given the variability in vancomycin PK, partially related to variability in residual kidney function (RKF) and dialysis prescriptions. Lexicomp recommends 10 mg/kg every 12 to 24 h, followed by TDM, though this is notably a lower total daily dose than the studies above suggest would be adequate for PD target attainment.

### Anti-virals

#### Acyclovir

Acyclovir is most used for HSV infections, which can be devastating in neonates. Acyclovir is mainly eliminated through the kidney. Given its small molecular size, low-protein binding, and water solubility, it is readily removed by CKRT [66]. Four case reports evaluated acyclovir PK in pediatric patients on CKRT [66–69]. The studies used a variety of dosing regimens (Table 1). Two studies demonstrated high acyclovir CL, requiring escalation in dosing, with one study ultimately putting acyclovir in the dialysate to achieve adequate levels [68, 69]. Another study reported significant intraindividual PK variability not only between each modality (CAVH, CAVHDF, and CVVHD), but also demonstrated decreased CL over time, even within the same modality [67]. Overall, this variability supports the use of TDM when possible. Current guidelines recommend 10 mg/kg/dose every 12 h for pediatric patients on CKRT.

### Anti-fungals

#### Fluconazole

Fluconazole undergoes substantial tubular reabsorption in patients with normal kidney function. As this reabsorption is absent in anuric patients on KRT, total CL in adults on CKRT has been demonstrated to be up to 2.3 times that reported in healthy volunteers [70]. One study evaluated fluconazole PK in a 17-year-old patient on high-effluent CVVHD [71]. They demonstrated significant CL of fluconazole and found the best dosing scheme after simulation to be 900 mg LD followed by 600 mg BID. The study suggested TDM be performed, due to inter- and intraindividual variability. Conversely, Lexicomp recommends 6–12 mg/kg only every 24 h (maximum 800 mg/dose). Additionally, TDM is recommended for critical illness, due to limited data and patient variability, with target trough concentrations of 10–15 mg/L.

## Hemodialysis (HD)

Pediatric HD catheter-associated bloodstream infections are common (3.3–30/100 patient months) and appropriate antibiotic treatment is important to prevent catheter loss [5]. Antibiotics may be administered before, during, or after dialysis, depending on their mechanism of efficacy and dialysis center practices [72]. Aminoglycosides are typically administered pre-HD to maximize peak (determinant of efficacy) and minimize trough (determinant of toxicity) concentrations, whereas other antibiotics are administered during or after HD. When TDM is performed, levels may be drawn before or after HD. When a patient is receiving care in an outpatient setting, intradialytic antibiotic administration ± pre-dialysis TDM is common, to minimize additional time a patient spends in the dialysis unit. We found only four studies of pediatric patients on HD, covering vancomycin, meropenem, and ceftazidime-avibactam (Table 2).

**Table 2:** Studies evaluating antimicrobial PK in pediatric patients on HD

Drug	Sample size	Age (y)	Wt (kg)	Patient population	Half-life (hours, on HD)	Half-life (hours, off HD)	Vd (L/kg)	PK goals	Dosing recommendation	Agreement with Lexicomp
Vancomycin [75]	16 patients (42 antibiotic courses)	Mean 8.17	Mean 26.08	Patients on chronic HD with presumed or documented infection	Median 2.73 (IQR 2.17–3.73)	Median 59.74 (IQR 38.04–69.62)	Mean 0.65	Trough 10–15 for central nervous system or MRSA coverage, 5–12 for others	10 mg/kg followed by TDM	Yes
Vancomycin [76]	1 patient	6	13.5	Anephric female with bacteremia	6.1	99.3	0.53	Peak 25–30, trough 10–15	15 mg/kg followed by TDM	No
Meropenem [74]	7 patients	Median 12 (range 1.4–17)	Median 36.8 (range 9.5–60.8)	Patients on chronic HD without active infection	Median 1.3 (range 1.1–1.7)	Median 7.3 (range 4.9–11.7)	Median 0.25 (range 0.12–0.29)	Concentration > 4 mg/L for 70% of the dosing interval	25 mg/kg daily or 40 mg/kg every other day (max 2000 mg)	Yes
Ceftazidime-Avibactam [73]	Simulated patients only								18.75 mg/kg every 48 h	Yes

Abbreviations: *y* years, *wt* weight, *HD* hemodialysis, *Vd* volume of distribution, *PK* pharmacokinetics, *MRSA* methicillin-resistant staphylococcus aureus, *TDM* therapeutic drug monitoring

### Beta-lactam antibiotics

#### Ceftazidime-avibactam

One study used combined adult and pediatric population PK models to develop ceftazidime-avibactam dose recommendations for pediatric patients with decreased kidney function, including those on HD [73]. However, only 1% of the 154 pediatric patients included in the model had decreased kidney function (< 50 mL/min/1.73 m^2^). Lexicomp recommendations are in concordance with the results of this study, and recommend 19 mg/kg every 48 h (based on the ceftazidime component) for patients with eGFR < 5 mL/min/1.73 m^2^, including those on HD.

#### Meropenem

One case series evaluated meropenem PK in 7 children on HD [74]. Meropenem CL on HD correlated with urea reduction, with a median of 81% CL with a single 3-h HD session. Dosing simulations demonstrated that a daily dose of 25 mg/kg or an alternate day dose of 40 mg/kg immediately after dialysis would result in an acceptable pharmacodynamic profile. Based on this study, the current meropenem recommendation for a pediatric patient on HD is a daily dose of 25 mg/kg or an alternate day dose of 40 mg/kg (maximum dose 2000 mg), with the dose being administered after HD on dialysis days.

### Other antibiotics

#### Vancomycin

Vancomycin is commonly used in patients on HD, both empirically and to treat documented infections, due to the high prevalence of MRSA [75]. Its long half-life (t_1/2_) in patients on dialysis allows for long dosing intervals. Two studies evaluated the PK of vancomycin in pediatric patients on HD. One study published in 1989 found that vancomycin CL was significantly increased by HD as demonstrated by the difference between t_1/2_ off (99 h) and on (6 h) dialysis [76]. This was a novel finding, as published studies in adults at that time had evaluated the CL of vancomycin using older dialyzer membranes, which could not as easily remove vancomycin. This study highlights the importance of continuing to evaluate the PK of drugs in HD as dialysis membrane efficiency improves over time. The second study included 16 patients and 42 courses of vancomycin [75]. This case series evaluated vancomycin concentrations before, immediately after, 1 h after, and 4 h after HD or hemodiafiltration (HDF). The mean vancomycin CL during HD was 56%, followed by a rebound phase during the 4 h post-dialysis, during which drug from the peripheral compartment came into the central compartment following HD, increasing concentrations. This resulted in an overall drug removal of 43%. Duration of dialysis and mode (HD vs. HDF) were significant predictors of PK parameters, with HDF providing higher CL. These studies recommended a LD of 10 mg/kg [76] and 15 mg/kg [75], based on their analysis. Both studies recommended pre-dialysis TDM in this population to inform maintenance doses due to variability in vancomycin elimination. The recommended initial dose in Lexicomp for vancomycin in a pediatric patient on HD is a LD of 10 mg/kg followed by TDM to determine maintenance dosing.

## Peritoneal dialysis (PD)

Antimicrobials are commonly used in patients on peritoneal dialysis for the treatment of both systemic infections and peritonitis. Peritonitis is a serious infectious complication for patients on PD and a major reason for PD failure and conversion to HD [77]. Intraperitoneal (IP) antibiotic administration is often the preferred method of treatment to allow for optimal antibiotic concentration at the site of infection and treatment at home. Swift and appropriate antibiotic treatment of peritonitis is important to protect the peritoneal membrane from prolonged inflammation and prevent refractory or relapsing peritonitis, which often require PD catheter removal. Most antibiotic PK studies have studied adults on continuous ambulatory PD (CAPD). This is as opposed to automated peritoneal dialysis (APD), which is more commonly used in pediatric patients. APD uses a machine to perform frequent, short exchanges of PD fluid overnight. CAPD, on the other hand, involves fewer, longer manual exchanges throughout the day. There is concern that extrapolating dosing from CAPD to APD could lead to underdosing and suboptimal antibiotic exposure [78]. The short frequent exchanges in APD may not allow enough time for systemic antibiotic absorption from IP administration and lead to increased CL and shorter t_1/2_. Systemic absorption is needed to ensure patients continue to have adequate antibiotic exposure when off PD in the daytime. For time-dependent antibiotics such as beta-lactams [25], significant CL may result in antibiotic concentrations falling below the bacterial minimum inhibitory concentration (MIC) for a significant portion of the dosing interval, leading to treatment failure. Conversely, acute peritonitis alters the permeability of absorption of drugs across the peritoneal membrane. This may lead to increased systemic absorption of drugs across the peritoneal membrane compared with volunteer PD patients without peritonitis, who were historically used as subjects in PK studies of IP antibiotics in patients receiving chronic PD [77]. Finally, neonates and young children receiving PD may have very low fill volumes, which may lead to inadequate antibiotic exposure with IP dosing or inadequate drug CL with IV dosing. Despite these significant concerns, there are scant PK data for antimicrobials in this population.

Although IP is the most common route of antibiotic administration in patients on PD, particularly when they are being treated for peritonitis, intravenous or enteral antibiotics may sometimes be used for a variety of reasons. For example, when there is concern for systemic sepsis, guidelines recommend using IV antibiotics, and enteral antibiotics may be used for mild infections such as pneumonia or acute otitis media [6]. Moreover, in recent years with concerns for fluid shortages, IV antibiotics may be preferred initially until cultures are confirmed to prevent large volumes of dialysis fluid with empiric, broad-spectrum antibiotics from being wasted. We found only 10 antimicrobial studies in pediatric patients on PD, covering cefazolin, vancomycin, and fluconazole.

### Cefazolin

A single case report described the pharmacokinetics of intravenous (IV) cefazolin in a pediatric patient on peritoneal dialysis [79]. Their study demonstrated relatively low PD CL. This study had significant limitations, as it was a single patient who was on PD for less than 48 h. There are no papers evaluating the PK of IP cefazolin in pediatric patients on PD. Current guidelines recommend 25 mg/kg/dose (maximum 1000 mg) IV cefazolin every 24–48 h or 500 mg/L IP cefazolin LD followed by 125 mg/L IP cefepime continuous maintenance dose when used for the treatment of peritonitis [6]. The same IV dose is recommended for non-peritonitis infections. These dosing guidelines were extrapolated from the adult literature and have not been evaluated in pediatric patients on PD. This is even though cefazolin is one of the most frequently used antimicrobial agents in pediatric patients on PD, with numerous indications in both prophylaxis and treatment guidelines [6].

### Vancomycin

Vancomycin is frequently used in pediatric patients on PD, especially in the treatment of gram-positive peritonitis [6]. Accurate vancomycin dosing is of particular importance in patients on PD, given the importance of preventing nephrotoxicity and preserving RKF. Preserved RKF in patients on chronic PD has been demonstrated to not only improve the adequacy of the KRT, but also accelerate the growth rate, improve nutrition, reduce the risk of adverse myocardial changes, and improve blood pressure control [80]. IP vancomycin PK has primarily been studied in adults on CAPD, with large variation in reported bioavailability (proportion of drug absorbed from the peritoneum into the bloodstream) ranging from 35 to 75% following a 4- to 6-h dwell [81, 82], which has been shown to increase during episodes of acute peritonitis [83].

Seven studies evaluated the PK of IP vancomycin in pediatric patients on PD. One study, which developed a PopPK model from 3 patients with 6 peritonitis episodes, reported that the current LD recommended for IP vancomycin (1000 mg/mL) was leading to excessive systemic vancomycin absorption, as defined by peak concentration > 50 mg/L, putting patients at risk for adverse effects [84]. PD bioavailability in this study was estimated to be 39% (95% confidence interval, 18–52%). The recommended LD in international guidelines was subsequently lowered (from 1000 to 500 mg/L), citing this study as rationale [6]. Another study evaluated the PK of IP vancomycin in children without peritonitis [85]. Vancomycin bioavailability in this study was 70%. These studies demonstrated the significant variability in IP vancomycin bioavailability that is seen, supporting the use of TDM. The other five studies used a variety of dosing strategies, as outlined in Table 3, and reported serum vancomycin concentrations during treatment, without doing any further PK analysis [86–90]. Current guidelines for the treatment of peritonitis recommend 500 mg/L IP vancomycin LD followed by a 25 mg/L IP vancomycin continuous maintenance dose [6]. For non-peritonitis infections, Lexicomp recommends 10 mg/kg/dose IV vancomycin, followed by TDM to further guide dosing. Given that IP administration of vancomycin results in systemic absorption, as evidenced by TDM, IP only treatment could be considered for a systemic infection. However, there are currently insufficient data to support this practice.

**Table 3:** Studies evaluating antimicrobial PK in pediatric patients on peritoneal dialysis

Drug	Modality (fill volume)	Sample size	Patient population	Age	Weight (kg)	Route	Dose used
Cefazolin [79]	Continuous (26 mL/kg)	1 patient	Clinical sepsis post cardiac surgery	16 months	7.5	IV	6 mg/kg every 8 h
Fluconazole [91]	Continuous (10–30 mL/kg)	8 patients	Clinical sepsis post cardiac surgery	2 weeks – 3 years (Mean 6 months)	2.8–5.7	IV	3 mg/kg daily
Fluconazole [93]	CCPD (340 mL)	1 patient	Stage 5 CKD with peritonitis	19 months	NR	IP	6 mg/kg LD (6-h dwell), then MD 3 mg/L. Repeat LD given based on levels
Fluconazole [92]	CCPD (40 mL/kg)	1 patient	Stage 5 CKD with peritonitis	5 months	4	IV	3 mg/kg daily over 1 h
Vancomycin [90]	Continuous (42 mL/kg)	1 patient	Stage 5 CKD with osteomyelitis	2 years	8.5	IP	MD 20 mg/L (had already received 2 IV doses)
Vancomycin [85]	CCPD and CAPD (1100 mL/m^2^)	7 patients	Stage 5 CKD without an active infection	5–17 (mean 12.7) years	25–89	IP	LD 500 mg/L
Vancomycin [84]	CCPD (35–40 mL/kg) (450—2000 mL, 1100 mL/m^2^ for model)	3 patients (6 peritonitis episodes)	Stage 5 CKD with peritonitis	3–16 years	13–48	IP	LD 500 mg/L or 1000 mg/L
Vancomycin [89]	Continuous (NR)	1 patient	Neonate with AKI and peritonitis	2 weeks	3.3	IP	MD 25 mg/L (no LD)
Vancomycin [88]	CCPD and CAPD (NR)	80 episodes	Stage 5 CKD with peritonitis	0.7–21.8 (median 11.4) years	NR	IP	Either (1) LD 15 mg/kg followed by MD 30 mg/L or (2) LD 30 mg/kg followed by second LD 7 days later
Vancomycin [86]	Continuous (NR)	1 patient	Peritonitis post cardiac surgery	3 months	4.2	IP	15 mg/L
Vancomycin [87]	CCPD (NR)	1 patient	Stage 5 CKD with peritonitis	11 years	50	IP	25 mg/L, then 50 mg/L, then 15 mg/kg q48 hours

*IV*, intravenous; *CCPD*, continuous cycling peritoneal dialysis; *CKD*, chronic kidney disease; *NR*, not reported; *IP*, intraperitoneal; *LD*, loading dose; *MD*, maintenance dose; *CAPD*, continuous ambulatory peritoneal dialysis

Of note, total dose delivered for IP administration depended on volume of dialysate, which was not always reported

### Fluconazole

Fungal peritonitis is a rare but devastating complication of PD. It is associated with high rates of PD discontinuation. Anti-fungal prophylaxis is recommended any time a patient on PD is receiving systemic or IP antibiotics for any reason due to an increased risk of fungal peritonitis. Fluconazole is the most used agent for anti-fungal prophylaxis, though it can also be used for the treatment of systemic or peritoneal fungal infections in patients on PD. Three studies evaluated the PK of fluconazole in 10 pediatric patients on PD. Fluconazole was administered intravenously in all but one patient, who received IP fluconazole. One study evaluated the PK of IV fluconazole in children recovering from major open-heart surgery, comparing patients on PD with patients who did not require PD [91]. PD effectively removed fluconazole and was the primary method of elimination in patients who required PD. CL was similar between the two groups, but patients on PD had a larger Vd, resulting in a significantly longer fluconazole t_1/2_. The other two studies reported fluconazole plasma concentrations only, without assessing the CL, t_1/2_, or Vd [92, 93]. International guidelines for the management of PD-associated infections in children recommend fluconazole 6 mg/kg IV or oral every 24–48 h (maximum 400 mg/dose) for treatment dosing [6]. This is twice as high as the IV dose used in the two studies we found (3 mg/kg IV daily). However, these studies considered a trough above MIC as their marker of adequacy, when the AUC:MIC ratio is most important for fluconazole, which has time- and concentration-dependent fungistatic activity, with a generally recommended 24-h AUC of 400 mg*h/L for treatment dosing [94, 95]. A 24-h trough concentration above the MIC would not necessarily guarantee adequate fluconazole exposure, implying a potential role for TDM.

## Conclusions

Proper drug dosing is important to maximize efficacy and minimize the risk of toxicity. Pediatric patients receiving either acute or chronic dialysis are at increased risk of infection, and may even be on dialysis for sepsis associated with AKI, but are underrepresented in studies evaluating antimicrobial dosing. This review identified multiple examples where previous dosing recommendations were changed after the drug was studied in the pediatric population [56, 74, 84]. Piperacillin dosing on CKRT was previously 100 mg/kg every 8 h, with the dosing interval increased to 12 h after the fourth dose. A PopPK created using samples from critically ill children on CKRT suggested a 24-h CI (200 mg/kg/day of piperacillin) would be optimal in this population, though 100 mg/kg every 8 h (30-min infusion) could be considered if a CI was not logistically possible [56]. Meropenem dosing in pediatric patients on HD was previously 20 mg/kg dosed after HD, until a 2001 study demonstrated that 25 mg/kg daily or 40 mg/kg dosed after HD was needed to maintain adequate bactericidal concentrations [74]. Finally, a 2021 study evaluating IP vancomycin in pediatric patients on PD demonstrated that the IP vancomycin LD should be decreased from 1000 to 500 mg/L to avoid excessive vancomycin peaks. Reassuringly, for the studies with sufficient data to provide generalized dosing recommendations [26, 30, 39, 41–47, 50, 55, 56, 60, 63–65, 73–76, 84], Lexicomp and ISPD dosing recommendations generally aligned with those from the primary literature (Supplemental Table 1). However, there are multiple antimicrobials which have no standardized pediatric dosing recommendations due to a lack of pediatric-specific data. This narrative review demonstrates that while there has been a push in recent years to better understand antimicrobial PK in children on dialysis, there continue to be major gaps.

Many of the studies found were single case reports or small case series, with findings that cannot be broadly applied. For combination drugs, the parent drug is typically studied, but not the beta-lactamase inhibitor. This further complicates drug PK for these drugs. Although variations in dialysis prescription and RKF can affect drug CL, existing drug PK data in pediatric patients on KRT is currently not robust enough to account for these variables in dosing recommendations. It is notable that multiple studies found even a small amount of residual UOP to impact drug clearance significantly [55, 61]. To better understand what factors may affect drug PK during various modes of dialysis, future studies should follow a minimum reporting dataset. These should include details regarding the presence of residual kidney function and specifics of dialysis prescriptions (e.g., blood flow rate, effluent flow rate, and filter used for CKRT; blood flow rate, dialysate flow rate, and filter used for HD; and fill volume, number of cycles, and ultrafiltrate volumes for peritoneal dialysis). An additional factor complicating drug dosing for critically ill patients on CKRT is the fact that they often receive adjunctive therapies (e.g., therapeutic plasma exchange, ECMO, or novel CKRT filters), which affect the patient’s CL and Vd. These additional therapies should be studied further, so that providers may feel more confident in antimicrobial dosing in this population.

It should be noted that studies for multiple antimicrobials (cefepime, vancomycin, fluconazole, and acyclovir) recommended TDM due to high PK variability and narrow therapeutic windows. However, we recognize that not all centers may have TDM readily accessible to them. This further underscores why more PK data in pediatric patients on KRT is needed, so that clinicians can feel more confident about initial dosing when TDM is not available or not readily accessible. Providers should also be aware of the PK variability that can exist when prescribing these antimicrobials, so they proactively monitor for efficacy and toxicity. Furthermore, when TDM is not available or delayed, using clinical pharmacology principles and knowledge of the PK drivers of efficacy can help guide dosing. For example, time over MIC is the major determinant for efficacy for beta-lactam antibiotics, so changing infusions to be extended or continuous may be justified when there is concern for inadequate antimicrobial effect.

Most of the studies evaluating antimicrobial PK in pediatric patients on dialysis have been in CKRT. While this population is important because these patients are critically ill, there is a large population of patients on HD and PD who are understudied. We found only 4 and 11 studies in pediatric patients on HD and PD, respectively. Even for commonly used drugs such as cefazolin, cefepime, and meropenem, which are considered first-line drugs for infections in these populations in international guidelines, there are little or no pediatric-specific PK data in pediatric patients on HD or PD. These drugs in these modalities must be prioritized for study. There were also several commonly used antimicrobials (amoxicillin, ampicillin, ceftaroline, ciprofloxacin, ganciclovir, metronidazole, and trimethoprim-sulfamethoxazole) without any pediatric PK data. Prescribers should be aware of the lack of data in this population, and think critically about the dosing when there is concern for treatment failure or toxicity. Even in the cases where there are data for any of the modalities, most of the studies were small, often case reports or series, and included heterogeneous populations with different diagnoses, indications for KRT and prescriptions. Thus, the confidence in the recommendations of these reports should likely be considered very low or low. These antimicrobial drugs must be prioritized for future studies in these populations, preferably through leveraging multi-center networks, such as WEROCK, a collaborative of centers for CKRT [23]. It is important to note that as technology and dialysis modalities evolve over time, we must continue to reassess how changes may affect drug PK. For example, in the 1990 s, it was believed that vancomycin was not dialyzable, until new studies demonstrated that the new high-flux dialyzers could more effectively remove moderate sized molecules, compared with older membranes [75].

In conclusion, antimicrobial PK in pediatric patients on dialysis are understudied, despite this population being at increased risk for infection and vulnerable to drug toxicity. Future research should focus on drugs that are most used and drugs with known/suspected risk of toxicity at elevated concentrations. Studies should evaluate how variables such as dialysis prescription and RKF affect drug PK and could be incorporated into dosing recommendations.

## Supplementary Information

Below is the link to the electronic supplementary material.

ESM 1 Graphical abstract (PPTX 157 KB)

ESM 2 (DOCX 41.2 KB)
