# Supplementary material for: Antimicrobial pharmacokinetics in pediatric patients on kidney replacement therapy: a comprehensive narrative review
Source: Pediatr Nephrol. 2025 Dec 6;41(8):2347–63. doi: 10.1007/s00467-025-07083-8 (PMC13337932; doi:10.1007/s00467-025-07083-8)
Supplement: Supplementary file 1 — Graphical abstract (PPTX 157 KB) [file 467_2025_7083_MOESM1_ESM.pptx]

## Slide 1
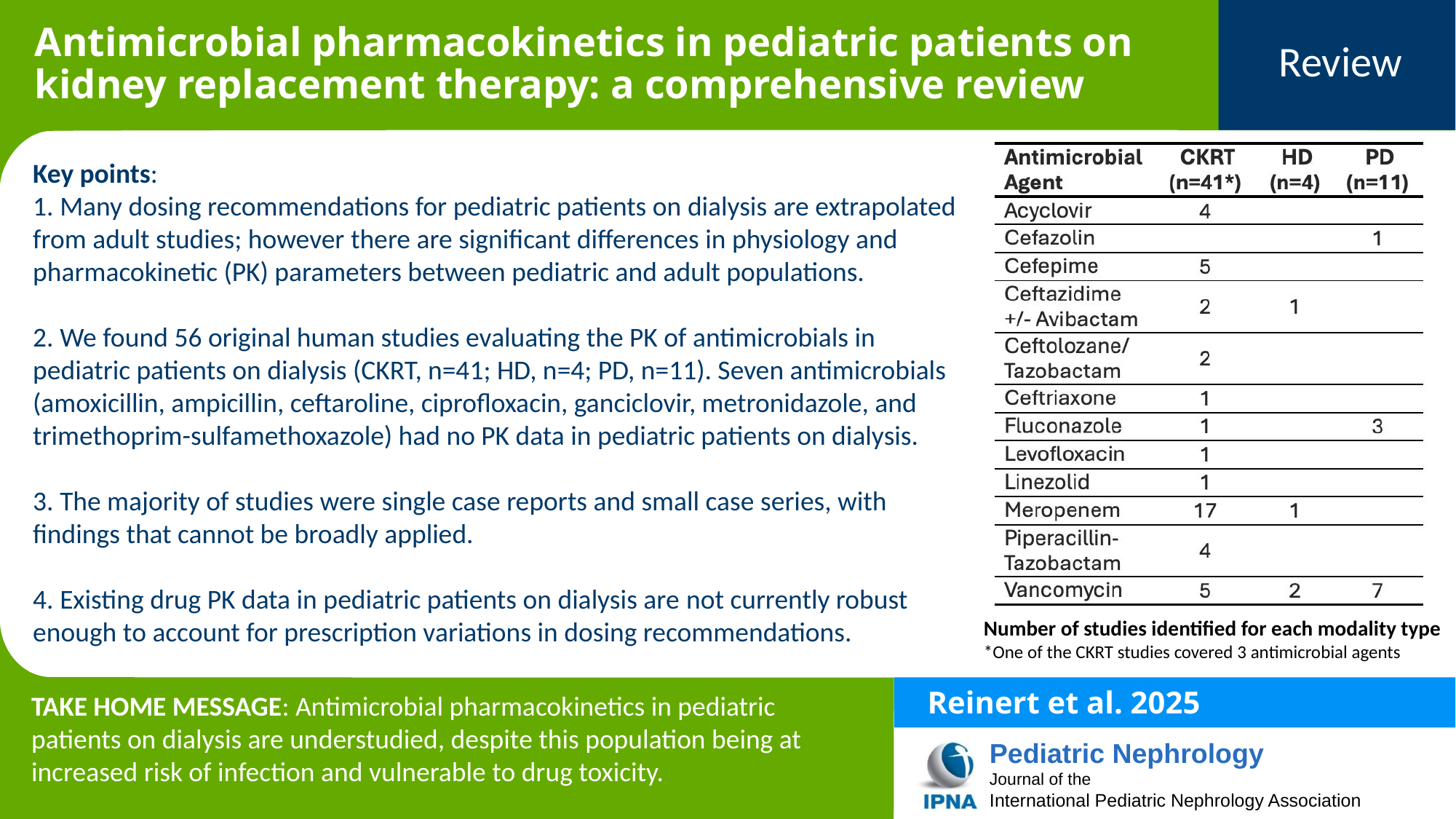

Antimicrobial pharmacokinetics in pediatric patients on kidney replacement therapy: a comprehensive review
Key points:
1. Many dosing recommendations for pediatric patients on dialysis are extrapolated from adult studies; however there are significant differences in physiology and pharmacokinetic (PK) parameters between pediatric and adult populations.
2. We found 56 original human studies evaluating the PK of antimicrobials in pediatric patients on dialysis (CKRT, n=41; HD, n=4; PD, n=11). Seven antimicrobials (amoxicillin, ampicillin, ceftaroline, ciprofloxacin, ganciclovir, metronidazole, and trimethoprim-sulfamethoxazole) had no PK data in pediatric patients on dialysis.
3. The majority of studies were single case reports and small case series, with findings that cannot be broadly applied.
4. Existing drug PK data in pediatric patients on dialysis are not currently robust enough to account for prescription variations in dosing recommendations.
Number of studies identified for each modality type
*One of the CKRT studies covered 3 antimicrobial agents
Reinert et al. 2025
TAKE HOME MESSAGE: Antimicrobial pharmacokinetics in pediatric patients on dialysis are understudied, despite this population being at increased risk of infection and vulnerable to drug toxicity.
