# Supplementary material for: Antimicrobial pharmacokinetics in pediatric patients on kidney replacement therapy: a comprehensive narrative review
Source: Pediatr Nephrol. 2025 Dec 6;41(8):2347–63. doi: 10.1007/s00467-025-07083-8 (PMC13337932; doi:10.1007/s00467-025-07083-8)
Supplement: Supplementary file 2 — (DOCX 41.2 KB) [file 467_2025_7083_MOESM2_ESM.docx]

Supplemental Table 1. Comparison of dosing recommendations for pediatric patients on RRT from PK studies and guidelines. References, when available and applicable, are provided in brackets.

| **CKRT** | **Primary Literature Recommendations** | **Current Lexicomp Recommendation** |
| --- | --- | --- |
| Cefepime | [1] Minimum 50 mg/kg q8h to achieve 100% *f*T>MIC, consider extended or continuous infusions for high effluent flows or resistant pathogens  [2] Doses > 50 mg/kg every 12 hours may be necessary | [2] 50 mg/kg every 8-12 hours (consider extended infusions or more frequent administration for certain situations) |
| Meropenem | [3] 20 mg/kg (over 4 hours) or 40 mg/kg (over 2 hours) q8h  [4] 20 mg/kg every 8-12 hours (depending on age)  [5] 20 mg/kg q12h  [6] 50 mg/kg/d (intermittent or continuous infusion depending on MIC)  [7] To reach 90% *f*T>MIC (MIC up to 2 mg/L)   - <10kg: 60-120 mg/kg/day CI is needed - >10 kg: 40 mg/kg q8h (30 min infusion) or 40 mg/kg q12 h (3-4 hour infusion) - >30 kg: 60 mg/kg/day CI is sufficient and avoids accumulation   [8] To reach 40% *f*T>MIC   - MIC up to 4 mg/L: 20 mg/kg q8h - MIC up to 8 mg/L: 60 mg/kg/day CI - Individual dose adjustments needed for 100% *f*T>MIC   [9] To reach 100% fT>MIC (MIC up to 8 mg/L)   - Qef <100 mL/hr: 20 mg/kg q12h - Qef 100-500 mL/hr: 20 mg/kg q8h or 20 mg/kg LD followed by 60 mg/kg/day CI - Qef 500-2000 mL/hr: 20 mg/kg LD followed by 60 mg/kg/day CI   [10] 20 mg/kg q8h (60-minute infusion) for MIC <1 mg/L would achieve 40-100% *f*T> MIC (depending on weight and kidney function) | [3, 6, 11] 20 mg/kg over 1-4 hours q8h.  40 mg/kg q8h may be necessary in some situations |
| Piperacillin | [12] 100 mg/kg q8h or 200 mg/kg/day CI  [13] 70-200 mg/kg LD followed by 140-400 mg/kg/day (divided in 2 daily doses or via CI). Dose dependent on MIC and residual diuresis | [12] 100 mg/kg q8h or 200 mg/kg/day CI |
| Linezolid | [14] Dose adjustment for KRT not needed | Dose adjustment for KRT not needed (based on adult literature) |
| Vancomycin | [15-17] 15-50 mg/kg/day divided in 2-3 daily doses, followed by TDM | 10 mg/kg every 12-24 hours followed by TDM |
| **HD** |  | **Current Lexicomp Recommendation** |
| Ceftazidime Avibactam | [18] 18.75 mg/kg q48h | [18] 19 mg/kg every 24-48 hours depending on residual kidney function |
| Meropenem | [19] 25 mg/kg daily or 40 mg/kg every other day  (max 2,000 mg) | [19] 25 mg/kg daily or 40 mg/kg every other day (max 2,000 mg) |
| Vancomycin | [20, 21] 10-15 mg/kg followed by TDM | 10 mg/kg followed by TDM |
| **PD** |  | **Current ISPD Recommendation** |
| Vancomycin (IP) | [22] 500 mg/L LD. No specific recommendations were given for MD in this study. | [22] 500 mg/L LD followed by 25 mg/L MD |

Abbreviations: *CI* Continuous infusion, *CKRT* continuous kidney replacement therapy, f*T > MIC* time free concentrations exceed minimum inhibitory concentration, *KRT* kidney replacement therapy, *LD* loading dose, *MD* maintenance dose, *Qef* total effluent flow rate, *TDM* Therapeutic drug monitoring

**Reference List**

1. Hambrick HR, Punt N, Pavia K, Mizuno T, Goldstein SL, Tang Girdwood S (2024) Monte Carlo simulations of cefepime in children receiving continuous kidney replacement therapy support continuous infusions for target attainment. J Intensive Care 12:38

2. Stitt G, Morris J, Schmees L, Angelo J, Akcan Arikan A (2019) Cefepime Pharmacokinetics in Critically Ill Pediatric Patients Receiving Continuous Renal Replacement Therapy. Antimicrob Agents Chemother 63:e02006-18

3. Tan WW, Watt KM, Boakye-Agyeman F, Cohen-Wolkowiez M, Mok YH, Yung CF, Chan YH (2021) Optimal Dosing of Meropenem in a Small Cohort of Critically Ill Children Receiving Continuous Renal Replacement Therapy. J Clin Pharmacol 61:744-754

4. Nehus EJ, Mouksassi S, Vinks AA, Goldstein S (2014) Meropenem in children receiving continuous renal replacement therapy: clinical trial simulations using realistic covariates. J Clin Pharmacol 54:1421-1428

5. Nehus EJ, Mizuno T, Cox S, Goldstein SL, Vinks AA (2016) Pharmacokinetics of meropenem in children receiving continuous renal replacement therapy: Validation of clinical trial simulations. J Clin Pharmacol 56:291-297

6. Rapp M, Urien S, Foissac F, Béranger A, Bouazza N, Benaboud S, Bille E, Zheng Y, Gana I, Moulin F, Lesage F, Renolleau S, Tréluyer JM, Hirt D, Oualha M (2020) Population pharmacokinetics of meropenem in critically ill children with different renal functions. Eur J Clin Pharmacol 76:61-71

7. Butragueño-Laiseca L, Troconiz IF, Grau S, Campillo N, Padilla B, Fernández SN, Slöcker M, Herrera L, Santiago MJ (2024) How to use meropenem in pediatric patients undergoing CKRT? Integrated meropenem pharmacokinetic model for critically ill children. Antimicrob Agents Chemother 68:e0172923

8. Pokorná P, Michaličková D, Tibboel D, Berner J (2024) Meropenem Disposition in Neonatal and Pediatric Extracorporeal Membrane Oxygenation and Continuous Renal Replacement Therapy. Antibiotics (Basel) 13:419

9. Thy M, Urien S, Bouazza N, Foissac F, Gana I, Bille E, Béranger A, Toubiana J, Berthaud R, Lesage F, Renolleau S, Tréluyer JM, Benaboud S, Oualha M (2022) Meropenem Population Pharmacokinetics and Dosing Regimen Optimization in Critically Ill Children Receiving Continuous Renal Replacement Therapy. Clin Pharmacokinet 61:1609-1621

10. Wang Y, Chen W, Huang Y, Wang G, Li Z, Yan G, Chen C, Lu G (2021) Optimized Dosing Regimens of Meropenem in Septic Children Receiving Extracorporeal Life Support. Front Pharmacol 12:699191.

11. Wang Y, Li Z, Chen W, Yan G, Wang G, Lu G, Chen C (2021) Pharmacokinetics of meropenem in children with sepsis undergoing extracorporeal life support: A prospective observational study. J Clin Pharm Ther 46:754-761

12. Butragueño-Laiseca L, Marco-Ariño N, Troconiz IF, Grau S, Campillo N, García X, Padilla B, Fernández SN, Slöcker M, Santiago MJ (2022) Population pharmacokinetics of piperacillin in critically ill children including those undergoing continuous kidney replacement therapy. Clin Microbiol Infect 28:1287.e9-1287.e15

13. Thy M, Urien S, Foissac F, Bouazza N, Gana I, Bille E, Béranger A, Toubiana J, Berthaud R, Lesage F, Renolleau S, Tréluyer JM, Benaboud S, Oualha M (2022) Piperacillin Population Pharmacokinetics and Dosing Regimen Optimization in Critically Ill Children Receiving Continuous Renal Replacement Therapy. Antimicrob Agents Chemother 66:e0113522

14. Yang M, Zhao L, Wang X, Sun C, Gao H, Qian S (2023) Population Pharmacokinetics and Dosage Optimization of Linezolid in Critically Ill Pediatric Patients. Antimicrob Agents Chemother 95:e02504-20

15. Vestal ML, Liu B, Heath TS (2023) Evaluation of Dosing Regimens of Vancomycin and Aminoglycosides in Pediatric Patients on Continuous Renal Replacement Therapy. J Pediatr Pharmacol Ther 28:143-148

16. Moffett BS, Morris J, Munoz F, Arikan AA (2019) Population pharmacokinetic analysis of vancomycin in pediatric continuous renal replacement therapy. Eur J Clin Pharmacol 75:1089-1097

17. Zylbersztajn BL, Izquierdo G, Santana RC, Fajardo C, Torres JP, Cordero J, Valverde C (2018) Therapeutic Drug Monitoring of Vancomycin in Pediatric Patients With Extracorporeal Membrane Oxygenation Support. J Pediatr Pharmacol Ther 23:305-310

18. Franzese R, Riccobene T, Carrothers T, Vourvahis M, Winter E, Lovern M, McFadyen L (2023) Population Pharmacokinetic Modeling for Ceftazidime-Avibactam Renal Dose Adjustments in Pediatric Patients 3 months and Older. Clin Pharmacol Ther 113:182-195

19. Goldstein SL, Murry DJ, May S, Aleksic A, Sowinski KM, Blaney S (2001) Meropenem pharmacokinetics in children and adolescents receiving hemodialysis. Pediatr Nephrol 16:1015-1018

20. Chung E, Tjon JA, Nemec RM, Nalli N, Harvey EA, Licht C, Seto W (2021) Pharmacokinetics of Vancomycin in Pediatric Patients Receiving Intermittent Hemodialysis or Hemodiafiltration. Kidney Int Rep 6:1003-1014

21. Schoumacher R, Chevalier RL, Gomez RA, Rogol AD, Cummings R, Spyker DA (1989) Enhanced clearance of vancomycin by hemodialysis in a child. Pediatr Nephrol 3:83-85

22. Hennessy K, Capparelli EV, Romanowski G, Alejandro L, Murray W, Benador N (2021) Intraperitoneal vancomycin for peritoneal dialysis-associated peritonitis in children: Evaluation of loading dose guidelines. Perit Dial Int 41:202-208
